# Comparison of two dual-energy CT-based methods for proton stopping-power ratio estimation in brain cancer patients with metal implants

**DOI:** 10.2340/1651-226X.2025.43930

**Published:** 2025-09-04

**Authors:** Ivanka Sojat Tarp, Vicki Trier Taasti, Maria Fuglsang Jensen, Ludvig Paul Muren, Kenneth Jensen

**Affiliations:** a Danish Centre for Particle Therapy, Aarhus University Hospital, Aarhus, Denmark

**Keywords:** Proton therapy, dual-energy CT, stopping power ratio, brain cancer

## Abstract

**Background and purpose:**

Accurate stopping-power ratio (SPR) estimation is crucial for proton therapy planning. In brain cancer patients with metal clips, SPR accuracy may be affected by high-density materials and imaging artefacts. Dual-energy CT (DECT)-based methods have been shown to improve SPR accuracy. This study evaluated the consistency between two SPR estimation methods in brain cancer patients: (1) a Hounsfield look-up table (HLUT) for DECT-generated virtual monoenergetic images (VMIs) and (2) the DirectSPR algorithm (Siemens Healthineers).

**Patient/material and methods:**

DECT scans were acquired for 11 brain cancer patients. Two SPR maps were generated: one using a 90 keV VMI with a HLUT and the other via the DirectSPR algorithm. The VMI HLUT was adjusted in high-density regions to align with the SPR of titanium. Clinically applied proton therapy plans were recalculated on both SPR maps and dose distributions were compared using dose-volume histograms. Furthermore, a voxel-wise SPR comparison and a separate titanium implant analysis were performed.

**Results:**

Dose differences between the SPR methods were minimal for organs-at-risk. DirectSPR showed strong SPR agreement with the VMI HLUT approach for CT numbers up to 1500 HU (SPR~1.9). Beyond this, especially in regions with titanium implants, DirectSPR yielded higher SPR values than the VMI HLUT, suggesting an adjustment may also be needed for DirectSPR.

**Interpretation:**

DirectSPR was consistent with the VMI HLUT up to 1500 HU but deviated at higher CT numbers. These deviations had limited impact on dose metrics, but they should be considered when choosing beam orientations.

## Introduction

Proton therapy is distinguished by its unique depth-dose distribution characterised by the Bragg peak [1]. This highly localised dose deposition limits the doses to the surrounding healthy tissue and thus has potential to reduce radiation-induced side effects [2]. The Bragg peak location is determined by the stopping-power ratio (SPR) of the tissues along the beam path, making accurate SPR estimation crucial for proton range prediction and dose delivery [3].

Computed tomography (CT) measures the X-ray attenuation in tissue, expressed as a CT number in Hounsfield Units (HU). The CT number must be converted into SPRs for use in proton treatment planning dose calculations [4]. Any uncertainties in this conversion, caused by differences between photon and proton interactions with human tissue, can introduce uncertainties in the dose deposited in the patient. Uncertainties in proton range estimation are accounted for by adding safety margins in the robust optimisation of the proton treatment plan [5]. However, this approach compromises the advantage of proton therapy as it limits the benefit of having no exit dose at the distal edge.

A common method for converting CT numbers to SPR values in clinical practice is to apply an empirical linear relationship between CT numbers and SPRs, denoted a Hounsfield look-up table (HLUT) [6, 7]. However, this approach does not allow for accurate estimation of all human tissues as in reality there is no one-to-one correspondence between CT numbers (governed by photon interactions) and SPRs (determined by proton interactions), and it also does not capture the natural variation between patients [3]. Advancements in imaging technology, particularly dual-energy CT (DECT), have improved SPR estimation accuracy [8, 9]. Unlike conventional single-energy CT (SECT), DECT acquires images at two different X-ray energy spectra, allowing better tissue characterisation within each voxel [10]. One way DECT can improve SPR estimation is through the creation of virtual monoenergetic images (VMIs), which simulate images obtained with a monochromatic X-ray beam [11]. This approach can reduce beam hardening and thereby improve the stability of the CT numbers, and thus the stability of the SPR estimate, while still using a HLUT as in conventional SECT [12]. Moreover, DECT also allows direct calculation of SPR using the Bethe formula by the estimation of material parameters derived from the high- and low-energy DECT images [13]. A few clinics have introduced such a direct SPR estimation approach based on DECT, which has reduced their clinical safety margins in proton therapy, improving treatment precision [14–16].

Patients undergoing postoperative proton therapy for brain cancer treatment may have surgically implanted metal fixation clips, which can cause artefacts in CT images. The artefacts occur due to the high density and atomic number of the metal, leading to beam hardening and streaking in the CT images which can compromise the accuracy of SPR estimation. This introduces uncertainties in proton range estimation and affects the precision of proton dose delivery [17, 18]. The aim of this study was to examine the consistency between two DECT-based SPR estimations: one using a HLUT for VMIs and the other obtained directly through a commercial DECT-based SPR algorithm [19], in proton therapy of patients with brain cancer.

## Patients/material and methods

### Patient cohort

This study included 11 adult patients with brain cancer (six female, five male; age range: 32–68 years). Ten of these patients were treated with postoperative proton therapy. Six of the treated patients had cranial fixation implants (CranioFix®). One untreated patient with a metal mesh implant was included in the study solely for the purpose of metal mesh analysis. All implants were made of titanium. The study was approved by Aarhus University Hospital institutional review board.

### CT scan settings

Each patient had two DECT scans, one acquired in split-filter mode (TwinBeam), and one acquired in sequential mode (Dual Spiral). The two DECTs scans were acquired consecutively, both on a SOMATOM Definition Edge CT scanner (Siemens Healthineers, Forchheim, Germany). The TwinBeam scan was used to create the clinical proton treatment plan, based on a VMI at 90 keV, following our clinical routine [20], but was not further used in this study. The Dual Spiral scan was acquired at tube voltages of 80 kVp and 140 kVp. The patients were fixated in thermoplastic masks, minimising movement between the TwinBeam and the Dual Spiral scan.

All CT images were reconstructed using iterative reconstruction (ADMIRE, strength 3) with Qr40 kernel and beam hardening correction for bone. An iterative metal artefact reduction algorithm (iMAR) was applied for patients with cranial titanium implants. The reconstructed CT images were 12-bit. The slice thickness was 1.5 mm.

### SPR map generation

Based on the Dual Spiral scan, two image sets were created using Siemens *syngo*. via (version VB50B_HF03): (1) a VMI at 90 keV applying the Mono+ algorithm [12] (for consistency, the same VMI energy was used as for the clinical TwinBeam scan), and (2) a SPR map applying the DirectSPR algorithm [19, 21].

The CT numbers from the Dual Spiral VMI (in the following simply referred to as VMI) were converted to SPR values by applying a HLUT created following a recent consensus guide [7]. This HLUT ensured correct SPR estimation for titanium, by artificially assigning CT numbers above 2950 HU (well above the CT numbers for human bone) to the SPR for titanium (see Figure 3, and Figure S1.7D in [7]).

The DirectSPR algorithm creates DICOM images with scaled SPR values as the voxel values. The SPR scaling is given as SPR_scaled_ = (SPR/1000)+1 [16]. This ensures that the DirectSPR images appear similar to a regular CT image.

For both the VMI HLUT and the DirectSPR, the SPR values were estimated at a proton energy of 100 MeV.

### Dose distribution analysis

For this study, the clinical treatment plan was recalculated in the Eclipse treatment planning system (version 16.1, Varian Medical Systems, Palo Alto, CA, USA) on both image sets from the Dual Spiral acquisition. For the VMI, the HLUT was applied, and for the DirectSPR map, a linear conversion curve was applied to descale the voxel values. In these dose recalculations, all planning parameters, such as beam geometry, monitor units, and the prescribed dose, were kept identical to the original clinical plan. Proton dose calculations assumed a constant relative biological effect (RBE) of 1.1. The dose distributions resulting from the VMI HLUT and DirectSPR-derived SPR maps were compared using clinically relevant dose-volume histogram metrics, including near maximum doses (D0.03 cm^3^), mean dose (Dmean), and the volume receiving 95% of the prescribed dose (V95%). Prescribed doses varied across patients: 36 Gy_RBE_ in 20 fractions (*N* = 1), 45 Gy_RBE_ in 25 fractions (*N* = 1), 50.4 Gy_RBE_ in 28 fractions (*N* = 1), 54 Gy_RBE_ in 30 fractions (*N* = 3), and 59.4 Gy_RBE_ in 33 fractions (*N* = 4).

### SPR comparison

SPR was compared between the VMI HLUT and DirectSPR approach by analysing SPR histograms within the body contour and the 20%-isodose region (ISO20; these volumes differed at most by 1% between the two SPR approaches), as well as within defined regions-of-interest (ROIs) containing titanium. Furthermore, the voxel-wise SPR values from the DirectSPR approach were plotted as a function of the corresponding CT numbers from the 90 keV VMIs and compared directly to the conversion curve defined by the VMI HLUT.

### Titanium implant analysis

To evaluate the impact of titanium implants under controlled settings, a titanium rod with a diameter of 1.5 cm was embedded in a Gammex Advanced Electron Density phantom (Sun Nuclear – A Mirion Medical Company, Middelton, WI, USA) and scanned in Dual Spiral mode using the same scanning and reconstruction parameters as for the patient scans. The SPR values were extracted from a cylindric ROI (1.0 cm diameter, spanning 27 axial slices with 1.5 mm slice thickness) that was placed entirely within titanium rod to avoid edge effects and partial volume artefacts.

The effect of titanium was also assessed in the patient data by analysing regions containing titanium implants, to determine the effect on the SPR accuracy and the resulting dose distribution. Seven of the brain cancer patients were included: six patients with titanium CranioFix clips, and one patient with a large cranial titanium mesh implant. The CranioFix implants were delineated using spherical ROIs (diameter = 1.5 cm), while the ROI for the cranial mesh was drawn freehand to account for its irregular shape (Figure 3). These ROIs also included areas of normal tissue. For the six patients with clips, a smaller ROI was also manually delineated to only include the titanium material (due to partial volume and image blurring, this is not an accurate process, which is the reason for also including the larger spherical ROIs).

To assess the potential worst-case impact of proton range uncertainty near the titanium implants, the manually delineated implant ROIs in the VMI images were overwritten with the SPR value of titanium, SPR = 3.1 for a proton energy of 100 MeV. The dose distributions were then recalculated for the VMI HLUT approach for the six patients with cranial titanium fixation clips.

## Results

### Dose distribution analysis

For the clinical target volumes (CTV), the V95% and Dmean were comparable between the two SPR methods, with a maximum difference of 0.1 percentage point (pp) for V95% and less than 0.06 Gy_RBE_ for Dmean (Table 1). The dose differences in the organs-at-risk (OARs) were also minimal between the two SPR methods, with no meaningful difference in mean or near-maximum (D0.03cm^3^) doses.

**Table 1:** . Volume of the clinical target volume (CTV) and absolute differences of dose-volume-histograms (DVH) parameters for the target and organs-at-risk (OARs). The differences were calculated by subtracting the DVH parameter values based on the DirectSPR approach from those based on the VMI HLUT approach. Entries marked as ‘NA’ (not applicable) denote that the respective organs-at-risk (OAR) received no dose, while an asterisk (*) denotes that the dose to the OAR was below 10 GyRBE. L/R: left/right.

DVH difference	CTV			Brainstem	Chiasm	Optical Nerve L	Optical Nerve R	Brain-CTV		Periventricular Zone
Patient number	Volume (cm^3^)	V95% (pp)	Dmean (Gy_RBE_)	D0.03 cm^3^ (Gy_RBE_)	D0.03 cm^3^ (Gy_RBE_)	D0.03 cm^3^ (Gy_RBE_)	D0.03 cm^3^ (Gy_RBE_)	Dmean (Gy_RBE_)	V30 Gy (cm^3^)	D0.03 cm^3^ (Gy_RBE_)
1	26.1	0.0	0.01	0.0	0.1*	NA	NA	0.0*	–0.5	–0.1
2	136.6	0.0	0.02	0.0*	NA	NA	NA	0.0*	–0.8	0.1
3	15.8	0.0	0.00	0.0	NA	–0.2	0.1	0.0*	–0.3	0.0
4	37.3	0.0	0.01	0.0	0.1	0.0	0.0	0.0*	–0.1	0.0
5	138.5	0.0	0.01	NA	NA	0.4	0.0*	0.0*	–1.0	–0.5
6	110.9	0.0	–0.01	NA	NA	NA	NA	0.0*	0.7	0.6
7	10.5	0.1	0.06	0.1	NA	NA	NA	0.0*	0.3	0.3
8	85.1	0.0	0.03	0.1*	NA	NA	NA	0.3*	0.0*	0.0
9	10.8	0.0	0.03	0.1	0.1	–0.4*	0.2	0.0*	–0.1	0.0
10	293.9	0.0	0.01	–0.7	–0.3*	0.0*	0.0*	-0.1	–1.9	0.1

### SPR comparison

**Figure 1:**
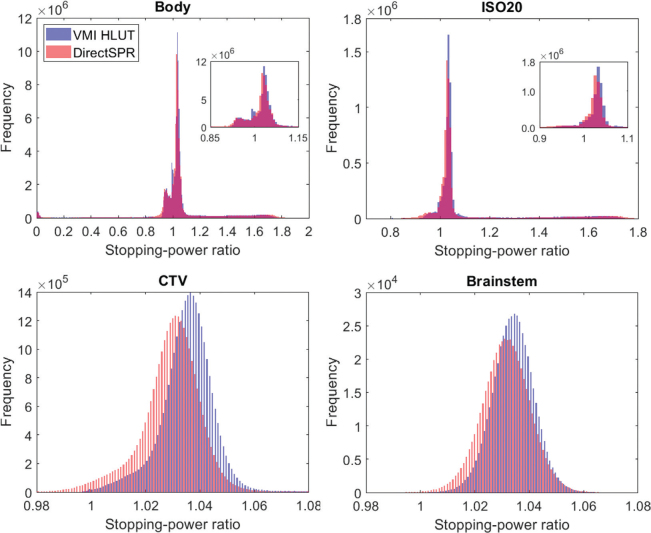
Histograms illustrating the distribution of SPRs across various anatomical structures derived from the VMI HLUT approach (blue) and the DirectSPR approach (red) including all 10 treated patients. SPR distribution within the Body contour (top left), the 20% isodose contour (ISO20; top right), the clinical target volume (CTV; bottom left), and the Brainstem (bottom right). For the Body and ISO20 contours, an inset with a zoom showing the peak near SPR = 1 is added.

Figure 1 shows the histogram analysis of SPR values across anatomical structures, comparing the VMI HLUT approach and the DirectSPR approach. The two methods show similar distributions for the body contour, with a dominant peak near an SPR of 1.03, corresponding to soft tissue, and a smaller peak at higher SPR values corresponding to dense materials, such as bone and titanium. Similarly, for the ISO20 contour, both approaches showed a narrow SPR distribution centred around 1.03, corresponding to soft tissue regions within the high-dose area.

For the CTV structure, the SPR histogram from the DirectSPR approach showed a slight leftward shift compared to the VMI HLUT, indicating consistently lower SPR values across the target region. The median SPR value for the VMI HLUT was 1.036 (standard deviation (SD): 0.019), whereas it was 1.030 (SD: 0.023) for DirectSPR, indicating a difference of 0.006 between the two methods. A similar trend was observed for the brainstem, where DirectSPR estimated slightly lower SPRs compared to the VMI HLUT, although the distribution shape remained consistent between the two methods. For the brainstem, the median SPR was 1.035 (SD: 0.008) for the VMI HLUT and 1.031 (SD: 0.010) for DirectSPR, with a difference of 0.004. For other relevant intracranial structures, only minor SPR differences were observed.

**Figure 2:**
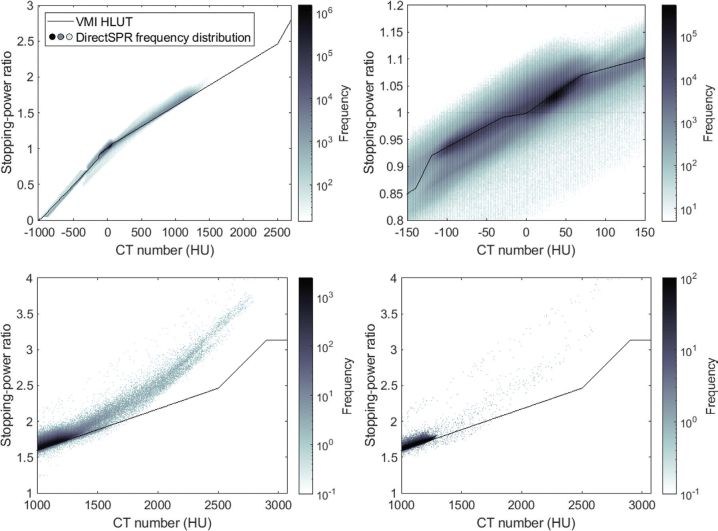
Frequency distribution of voxel-wise relationship between CT numbers for the VMI at 90 keV and SPRs from the DirectSPR approach for all voxels within the body structure across the 10 treated patients. For the VMI HLUT approach, the SPR values all follow the conversion curve shown by the black line, while for the DirectSPR approach, the SPR distribution is given by the coloured density map. Full CT number range illustrating the overall distribution (top left). Zoom-in of the soft tissue region (top right). High-density region including bone and titanium implants (bottom left). High-density region specifically within the ISO20 structure (bottom right). The grey colour scale represents the frequency distribution on a logarithmic scale, with darker shades representing higher frequency.

The voxel-wise comparison of the two SPR maps (Figure 2) showed the consistency of the two methods but also visualised the inflexibility of the VMI HLUT approach. For the DirectSPR approach, the SPR values were plotted as a frequency distribution and overlaid on the one-dimensional VMI HLUT. For CT numbers below 1500 HU, the SPR values from DirectSPR were scattered closely around the VMI HLUT, with the high frequency region on top of the VMI HLUT. This agreement was especially strong for soft tissues where the DirectSPR frequency map showed a dense concentration of data points close to the VMI HLUT. However, above 1500 HU, discrepancies became increasingly evident, particularly in titanium regions, where DirectSPR yielded higher SPR values than the VMI HLUT.

### Titanium implant analysis

**Figure 3:**
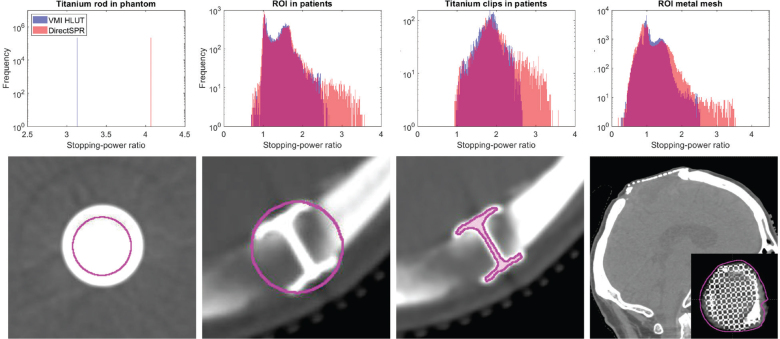
SPR estimation in the presence of titanium implants using the VMI HLUT approach and the DirectSPR approach. *Top row*: Histograms of the voxel-wise SPR values within each region-of-interest (ROI) comparing the VMI HLUT approach (blue) against the DirectSPR approach (red). For the CranioFix clips, the histograms include all six patients. *Bottom row*: Example CT images showing the ROIs (magenta contours) around various titanium implants used for analysis. *From left to right*: Axial slice of a titanium rod inserted in the Gammex phantom; axial slice of CranioFix titanium clips in a patient with a spherical ROI around the implant; axial slice of CranioFix clips in a patient with manually delineated ROI; oblique slice of a patient scan with a cranial titanium mesh implant (patient not treated).

Figure 3 shows examples of CT images illustrating analysed ROIs delineated around titanium implants in both the phantom and patient datasets. In all cases, systematic differences were observed between the VMI HLUT and the DirectSPR for SPR estimation in the presence of titanium. In the VMI HLUT method, the conversion curve was explicitly defined to assign an SPR of 3.1 (corresponding to SPR for titanium for a proton energy of 100 MeV) to all voxels with CT numbers above 2950 HU (see Figure 2), successfully encompassing all voxels within the titanium rod, whereby the estimated SPR matched the true SPR. However, the DirectSPR method estimated a higher SPR value of 4.1 for the ROI in the titanium insert.

For the patients, the SPR histograms for the DirectSPR method were shifted towards higher SPR values across all ROIs containing titanium, with broader distributions compared to the VMI HLUT method. Although the VMI HLUT was adjusted in the high-density region to reflect the SPR of titanium, none of the implant voxels exhibited CT numbers consistent with pure titanium, likely due to partial volume effects. For the spherical titanium ROIs, the median SPR was 1.436 (interquartile range [IQR]: 0.501) for the VMI HLUT and 1.462 (IQR: 0.549) for DirectSPR, and for the ROI covering only the titanium clips, the median SPR was 1.845 (IQR: 0.342) for VMI HLUT and 1.894 (IQR: 0.474) for DirectSPR. For the cranial mesh implant, the median was 1.018 (IQR: 0.328) for VMI HLUT and 1.001 (IQR: 0.436) for DirectSPR. That is, DirectSPR generally showed higher median SPR values and broader distributions than the VMI HLUT.

Table 2 shows the dose impact of manually overwriting the SPR values of high-density structure in the VMI HLUT approach, using the correct titanium SPR value of 3.1. The CTV V95% remained at 100% in three of the six patients. In patient 5, the target coverage decreased to 97.1% when overwriting the titanium clips (note, no overwrite was performed in the original treatment plan, but the HLUT for the TwinBeam VMI had a similar trend as for the Dual Spiral VMI HLUT used here). This was associated with a localised dose decrease of approximately 8 Gy_RBE_ in one field, located distal to the implant. The number of overwritten voxels ranged from 237 to 1611 (0.05–0.21% of the ISO20 volume). Within the ISO20 volume, the proportion of voxels with SPR > 1.9 (corresponding to a CT number of 1500 HU in the VMI HLUT) was slightly higher for the DirectSPR compared to VMI HLUT, but differed at most by 0.06 pp. The number of voxels with SPR > 1.9 remained below 0.13% in all patients.

**Table 2:** Impact of overwriting titanium implant with the correct SPR for titanium on the CTV V95% for the six treated patients with titanium clips. The proportion of voxels within the 20%-isodose region (ISO20) with SPR > 1.9 (corresponding to CT numbers > 1500 HU in the VMI HLUT-approach) and the number of voxels in the ISO20 volume manually assigned an SPR of 3.1 to represent cranial titanium clips are also listed. HLUT_Ti is the HLUT approach with a manual overwrite of titanium.

Patient	CTV V95% (%)			%voxel in ISO20 with SPR > 1.9		Overwritten voxels	
	HLUT	HLUT_Ti	DirectSPR	HLUT	DirectSPR	#voxels	%
2	100.0	98.7	100.0	0.07	0.09	1,203	0.10
5	100.0	97.1	100.0	0.03	0.06	771	0.08
6	100.0	99.9	100.0	0.07	0.06	841	0.09
8	100.0	100.0	99.9	0.07	0.13	237	0.05
9	100.0	100.0	100.0	0.00	0.01	847	0.21
10	100.0	100.0	100.0	0.04	0.09	1,611	0.10

## Discussion and conclusion

This study evaluated two DECT-based approaches for estimating SPR: the HLUT method applied to VMIs at 90 keV, which relies on an empirical conversion curve, and the DirectSPR algorithm, which estimates SPRs directly from DECT data, offering a more patient-specific approach. On average, the two methods showed strong agreement for tissues with CT numbers below approximately 1500 HU, covering most regular tissues in brain cancer patients. Note, the consensus guide followed here to create the VMI HLUT was already defined to ensure consistency between these two approaches (see Figure S1.6 in [7]). This study can thus be seen as validating the consensus guide HLUT creation in our brain cancer patients. Whereas the VMI HLUT is a one-to-one conversion offering limited flexibility, the DirectSPR approach better accounts for patient-to-patient variation (Figure 2).

Our findings suggest that the two SPR estimation methods yield clinically comparable results. This supports the use of DirectSPR as a potential alternative to VMI HLUT methods in clinical workflows. However, for CT numbers above 1500 HU discrepancies became more pronounced, particularly in regions containing titanium implants, where the DirectSPR algorithm tended to estimate higher SPR values than the VMI HLUT approach (Figure 2). Still, our dose analysis revealed only minimal differences between the two SPR estimation approaches regarding target coverage and dose to OARs (Table 1), since the beam arrangements were selected to avoid traversing the titanium implants when possible. This suggests that high-density materials need appropriate consideration, when using the DirectSPR algorithm.

Metal implants, such as titanium clips and meshes, pose challenges for accurate SPR estimation in proton therapy due to their high atomic numbers and associated image artefacts. This was seen despite the application of a dedicated metal artefact reduction algorithm to patients with titanium implants, which has previously been shown to improve range calculations in proton therapy [17].

The VMI HLUT approach was forced to assign an SPR of 3.1 for CT numbers above 2950 HU, matching the expected SPR of titanium. This ensured a correct SPR estimate for all titanium voxels in the phantom rod (Figure 3). In contrast, the DirectSPR method estimated an SPR of 4.1 in the same region, an overestimation of 30%, highlighting its limitations in the high-density regions.

To explore the worst-case clinical effect of SPR inaccuracies, implant voxels were manually overwritten with SPR = 3.1 in six patients with cranial clips. While this likely overrepresents the titanium structure due contouring and resolution limitations, it allows evaluation of the maximum clinical effect. Under these conservative assumptions, only one patient (Patient 5) experienced a clinically relevant reduction in CTV coverage (V95% < 98%) from a suboptimal beam direction intersecting the implant. In the remaining patients, the dose impact was negligible. DirectSPR did not outperform VMI HLUT in this context, as it lacks a dedicated correction for titanium implants. An SPR conversion scheme with a horizontal segment for high CT values (as in VMI HLUT implementation) could improve the performance of DirectSPR in the presence of titanium. However, with ≤ 0.21% of voxels containing titanium, the overall dose impact is likely minimal. These results suggest that while partial volume effects and uncorrected metal artefacts can compromise SPR accuracy, their clinical impact is often minor. Nevertheless, in patients where beam paths intersect metallic implants, care should be taken during plan evaluation, and conservative approaches such as SPR overwriting may be justified in selected cases.

Studies have shown that DECT provide more accurate SPR estimation than SECT [22–24]. A recent study by Yagi et al. also showed the benefit of DECT for SPR estimation in porous human bone and an improved dose calculation accuracy in heterogeneous regions like the skull base [25]. Taasti et al. [8] compared SECT and DECT for SPR estimation in proton therapy for head-and-neck cancer patients, finding that DECT-based methods could lead to SPR differences of the order of 1%, which may be clinically relevant for tumours adjacent to critical structures. Integrating DirectSPR into clinical workflows offers the potential to eliminate site-specific HLUTs and allows for the direct use of patient-specific physical parameters to estimate SPR [9]. Peters et al. reported that implementing DECT-based direct SPR estimation enabled the reduction of clinical safety margins from 3.5% to approximately 2%, resulting in 2.6–4.4 mm of healthy tissue sparring in the beam direction [14]. Similarly, Taasti et al. found that decreasing range uncertainty from 3% to 2% lowered OAR doses in 89% of neuro-oncological patients, with 44% also experiencing reduced toxicity [16]. A comparable dose-sparing effect was reported in a study involving 30 brain cancer patients, where reducing range uncertainty from 3.5% to 2.0% led to a median reduction of 4 cm³ in the volume receiving 80% of the prescribed dose [20]. These studies and our current findings underscore the clinical value of improved SPR estimation offered by DECT and DirectSPR.

The absence of a definitive ground truth for SPR in vivo limits the ability to assess which of the two methods is more accurate in the patients. One technical limitation in our study is the use of 12-bit DECT scans, which cannot fully represent highly attenuating materials like metals. Note for example that the CT number threshold of 2950 HU used to introduce the horizontal segment in the VMI HLUT for titanium SPR should be reconsidered for 16-bit images. Another challenge is the partial volume effect, where a single voxel contains a mixture of different tissue types [26]. In such cases, the CT number represents an average of the constituent materials, potentially leading to incorrect SPR estimation. This can be particularly problematic for small, high-density materials like titanium cranial fixation, as their small size can result in an underrepresentation of their actual density, which may lead to an underestimated SPR value in the voxel. In this study, although the VMI HLUT was adapted to reflect the SPR of titanium, observed CT numbers in the high-Z regions remained below the expected values, resulting in a median SPR of 1.845 (IQR: 0.342), which is notably lower than the expected value of 3.1.

In conclusion, the VMI HLUT and DirectSPR methods showed good agreement for SPR estimation in tissues with CT numbers below 1500 HU, including soft tissue and bone. However, notable differences were seen in high-density regions, particularly for titanium implants. DirectSPR consistently yielded higher median SPR values and broader distributions compared to VMI HLUT, with values closer to the expected SPR of titanium. While these differences had limited effect on dose distribution in our patient cohort, they may become clinically relevant if proton beams intersect metallic implants. These findings support the clinical feasibility of DirectSPR in brain cancer patients, provided appropriate handling of metal implants.

## Data Availability

The participants of this study did not give written consent for their data to be shared publicly, so due to the sensitive nature of the research sup-porting data are not available.
